# A conversation with Katherine High

**DOI:** 10.1172/JCI170663

**Published:** 2023-04-17

**Authors:** Ushma S. Neill

Physician-scientist and gene therapy pioneer, Dr. Katherine High, had a long career as an academic hematologist studying hemophilias and gene therapy vectors, before moving into industry. In both realms, High (Figure 1) played a massive role in bringing forward the first FDA-approved gene therapy. To hear more about isolating and cloning genes before PCR and whether she has a future in politics, see the full interview on www.jci.org/videos/cgms.

*JCI*: What were you like as a child?

High: I read a lot of books, and I really liked math. Some of my early memories are of doing assignments from my dad, like writing from 1 to 1,000. I’d made the mistake of thinking that after 100 comes 200 and then 300 and then 400 and was rewarded by having to write out all the numbers between 100 and 1,000. I played the piano and the viola, and I was on the swim team.

My dad worked in marketing for a very large photographic studio in the Southeast, which did the catalogs for Sears, JCPenney, and Montgomery Ward. Most of his business was in New York and Chicago, but he would not move there, and so we lived in North Carolina. My mother mostly worked at home. She also did some editorial work as we got older.

*JCI*: At age 10 you got a chemistry set from Santa Claus, and that began a lifelong love of chemistry?

High: I was the oldest of three daughters, and my dad was determined that he would get a scientist or an engineer out of one of us. Santa Claus did bring me a chemistry set, and it had all the chemicals you needed to do about a hundred different experiments. That was indeed the beginning of my introduction to science. I found chemistry more interesting than physics or biology, so despite a lot of pressure from my dad, who was hoping I would go into aeronautical engineering and then go work for NASA, I stuck with chemistry and headed to Harvard for my AB.

I really did enjoy majoring in chemistry, but when I was a senior, I thought maybe I should consider medical school and should probably take one year of biology and liked it. After I graduated, I took a job working in a pathology lab at the MGH in a research group that wanted me to synthesize oxidized derivatives of homocysteine. They injected those into rabbits to see if they could recapitulate the advanced atherosclerosis that occurs in people with homocystinuria. We were trying to figure out what the active metabolite was. It served as a good introduction to research and medicine, so I applied to medical school.

After the first two and a half years of medical school, I decided to take some time off and return to a chemistry lab. I found a great position in a polymer chemistry lab. I concluded, after I’d been there for about six months, that I should leave medical school and go to graduate school in chemistry. I went to talk to the head of the lab, and he told me that no one would care what terminal degree I had, and that the fastest option would be to finish medical school and then do a postdoc.

That made a lot of sense; I went back to medical school with the intent to finish up and then do as he suggested. But the rotations that I had when I got back were the ones that I turned out to like the most: pediatrics and medicine. I shifted my goals again and decided to finish medical school and do training in internal medicine.

*JCI*: Why turn toward hematology?

High: I wanted to go into a subspecialty where I could use my chemistry background and narrowed the choices to endocrinology, hematology, or rheumatology/immunology, as, at that time, those subspecialties were best understood at the molecular level. Then, I considered which patients I liked the most when on the medicine service, and that was how I decided for hematology.

I matched at Yale and joined Ed Benz’s lab, but there was also excitement in Bernie Forget’s lab, the division chief at that time, around mutations in the globin gene and the molecular dissection of the thalassemias. Most of my work focused on the *MYC* oncogene, but during that time, the genes for factor VIII and factor IX were isolated and characterized. I realized that it would be possible to explore the molecular basis of the hemophilias in the same way that people had already done for thalassemias, enabled by the cloning of those genes. That was the jumping off point for my independent career.

I took my first faculty job at UNC Chapel Hill. They had a large research operation in blood coagulation that spanned the gamut from chemists who worked on the structure of clotting factors, to biochemists who were purifying factor VIII from plasma, to a hemophilia center with abundant patient material. We delineated defects in people with hemophilia B, because the gene was smaller and was a little more tractable. The second project I worked on was defining the defect in the hemophilia B dog colony at Chapel Hill. I started by doing low stringency screens of a cDNA library made from canine liver. We also expressed mutant proteins and did structure function studies. And we began to do experiments with gene transfer into cells. Part of our motivation was that during that period, the late 1980s, the consequences of HIV infection were becoming obvious in the hemophilia population.

This was the largest hemophilia clinic on the East Coast, and we knew early on that nearly all the severe hemophilia patients A and B were HIV positive, but nobody knew what that meant. Our friends in infectious disease told us most viral diseases are not fatal, but, of course, that turned out not to be the case for HIV. We did not have any antiretroviral agents at that time, and after a long time we had AZT, which, as a single agent, was of limited utility. I was attending on the clinical coagulation service for three months a year, and the service became overwhelming as the number of hemophilia patients with AIDS in the hospital increased.

During my attending months, it became very difficult to manage anything that was going on in the research lab, to say nothing of the fact that it was heartbreaking. I received a job offer from Penn, and it was in the Department of Pediatrics, and while I’m not a trained pediatrician, my clinical responsibility was to be running the hematology and coagulation laboratories, which is a much more manageable responsibility while also supervising a research laboratory.

*JCI*: Your research evolved over this time, but when did you start to fixate on gene therapy?

High: My major motivation was still AIDS in the hemophilia population; I kept thinking I wanted to give these patients their own gene to make clotting factor themselves. Then they wouldn’t have to worry about hepatitis C or HIV from plasma-derived products. It was while I was at UNC that I first began to think about this and wanted to develop vectors that we could use to treat the hemophilia dog model, to then apply that pathway to humans.

At Penn, Bill Kelley was the dean, and he was aggregating resources and people around gene therapy, and I thought this could really help our efforts. At the beginning, we were agnostic about the vector; we made adenoviral and AAV vectors and wanted to see what worked the best. The adenoviral vectors triggered a brisk immune response even in mice, so we moved forward with AAV. We were able to show in 1997 that we could cure hemophilia in a mouse using an AAV vector, and in 1999, we published our first paper describing a cure for hemophilia in dogs.

It was difficult to make enough AAV in those days to treat a 20 kg dog, and the only people who were able to do that were at gene therapy companies. One, Avigen, was interested in our program and had already spent a lot of their resources streamlining manufacturing. We started collaborating, and they made the vector to do the experiments in the hemophilic dogs. After our success, we thought we could just move it into humans.

It was a longer story than that. The first human trial of AAV in skeletal muscle showed the muscle could make factor IX, but we were never able to get adequate circulating levels in an adult male the way that we did in dogs. It was safe, so we tried the liver, where even in the dog, you get higher circulating levels because hepatocytes secrete what they’re making directly into the blood. We got a dose that worked beautifully in the dogs and saw great levels of expression in the first patient who got that dose.

After about six weeks, the levels in that patient started to slowly decline, and they eventually disappeared. That was such a difficult experience even for us in the lab, and for the patient, I cannot imagine. This patient was a physician himself, and he talked about that; it was very disappointing for him. Another hemophilia patient described it once as the sensation of touching a rainbow.

*JCI*: And then had to go back into the rain.

High: Exactly. We spent the next several years trying to develop the reagents that we needed. We hypothesized that we had not seen an immune response in dogs or mice because they are not natural hosts for AAV, which humans are, and that humans probably had some memory response to AAV that was gumming up the works. We proposed that a short course of immunosuppression would allow the AAV capsids to be degraded and cleared from the cell, and then the patient should maintain durable expression.

To the extent that we could lower the dose, we could reduce the risk of an immune response. Eventually, we found a naturally occurring variant of factor IX, a high specific activity variant called factor IX Padua from a kindred from Italy where the proband had a factor IX level of 770%. His factor IX gene had a single-point mutation that made a much higher specific activity variant. We put that into a capsid that we had developed that was really hepatotropic and were able to lower the dose of the vector. Patients either had no immune response or one that was easily controlled with a short course of steroids. That was the basis of an exciting phase I/II trial. By the time that happened, we had already formed Spark.

To backtrack a little bit, in the late 1990s and early 2000s, there were some high-profile adverse events in gene therapy. They were not in the work we were doing. I should have realized that they were likely to affect all of gene therapy. The climate around us was becoming very skeptical, and eventually, the company that made our vector for the dog studies and then for our early-phase clinical testing could not raise money to work on gene therapy anymore. The only way that we could keep going is if I could find some other source of clinical grade vector.

*JCI*: You’re being very diplomatic, as some of these high-profile events happened at Penn. What kind of power of persuasion must you have had to get Penn to invest in a vector development program in the wake of that?

High: One of the most surprising events of my career was that success. When I went to the CEO of Children’s Hospital, who is a physician himself, I told him we needed to set up vector production in the hospital, or all our research efforts would die. At that point, there were headlines in the *Wall Street Journal* that said things like, “Gene therapy: Cursed or inching towards credibility?” As I walked out of his office, I was thinking about what my plan B could be.

A week later, and to my great surprise, his answer was yes, but I could not spend all the money on hemophilia. We had to work on other diseases that affect the pediatric population. Because I had long admired Jean Bennett’s outstanding data in a dog model of a form of congenital blindness, I asked her if she would work with us on a trial that we could do at Children’s Hospital, wherein we would produce the vector and we would work on getting the clinical trial approved through all the regulatory hurdles that gene therapy had to go through then, which included not only the FDA, but also the NIH Recombinant DNA Advisory Committee. In 2005, we started working on that together. In 2007, the clinical trials started, and even at the beginning, it seemed clear that a therapeutic effect was occurring. When you put very small doses of AAV into a relatively immunoprivileged space like the subretinal space, there are fewer concerns about the immune response, and we didn’t find one even when we went looking.

We had a very collaborative relationship with the Office of Cell and Gene Therapy at the FDA, but there were a lot of hurdles. There had never been a pharmaceutical treatment for inherited retinal dystrophies, and so we had to develop an endpoint, in addition to all the gene therapy hurdles. After all the early positive data suggested that it was working, we began the phase III trial in 2012. And then, that same CEO of the hospital called me in and asked what we would do if it worked — because CHOP is a hospital and wasn’t in the game to commercialize products.

*JCI*: Is this the reason you left Penn to start Spark Therapeutics?

High: He formed a subcommittee of the board to look at options. I was getting cold calls in my office from investors, and we were also getting inquiries from large pharma and big biotech that were focused on rare disease. In the end, I was worried about putting this program into a large company that was not focused entirely on gene therapy, because I was afraid that they would encounter some other problem that we had not already solved, and that they wouldn’t want to put additional resources into the program for this relatively small indication. In the end, we felt the safest thing to do was to put it into a gene therapy company that we would start.

*JCI*: Did you and Jean have a giant bottle of champagne when the approval came in for Luxturna, the first FDA-approved gene therapy for a genetic disease?

High: Well, first was the advisory committee meeting. The FDA doesn’t do this for every drug that they approve, but sometimes they assemble a panel of experts, and the sponsors have to present their data and go through what feels like an all-day oral exam on the data and the implications. We had a presentation that probably had 100 slides in it, with 900 backup slides. I was chairing for the sponsor; around 3:30 pm, the chair of the advisory committee declared there had been adequate discussion, and it was time to vote. I remember sitting down, and I was going to start crying because I thought, “Wow, I’ve been working on this for 12 years, and now my fate is going to be determined by something I can’t control.” It was a unanimous vote in favor of approval. That was really, really exciting. I was exhausted, and most of my Spark colleagues went back from Washington to Philadelphia on the bus, and the bus had champagne, but I went back in a car so that I could take calls from reporters.

*JCI*: At the end of a long day, do you ever think about what alternative career path you could have taken?

High: I always enjoyed reading. Perhaps, I would have been a teacher.

## Figures and Tables

**Figure 1:**
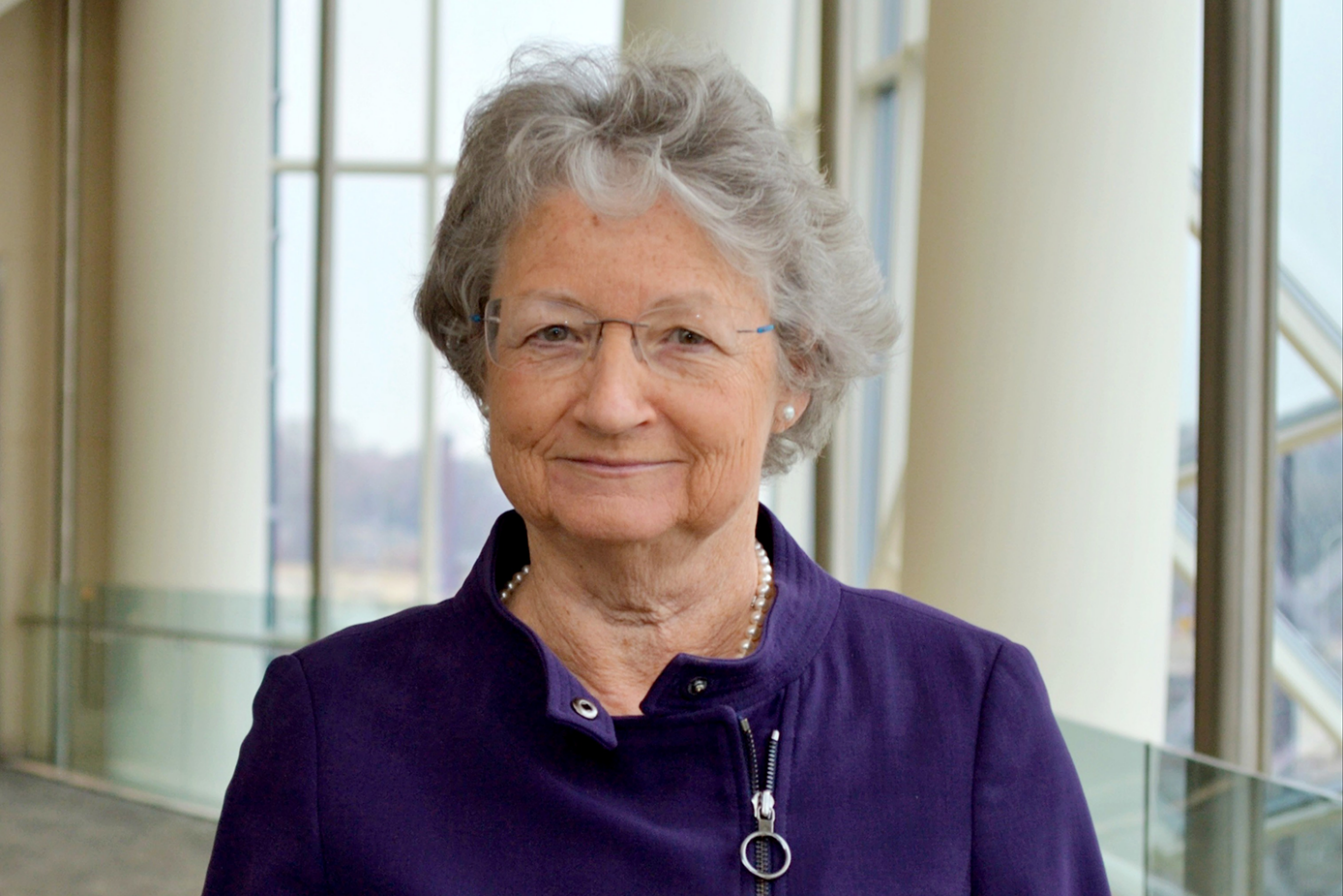
Katherine High.

## Version 1 04/17/2023

Electronic publication

